# Stable producer–scrounger dynamics in wild birds: sociability and learning speed covary with scrounging behaviour

**DOI:** 10.1098/rspb.2016.2872

**Published:** 2017-04-12

**Authors:** L. M. Aplin, J. Morand-Ferron

**Affiliations:** 1 Edward Grey Institute of Field Ornithology, University of Oxford, Oxford OX1 3PS, UK; 2 Department of Biology, University of Ottawa, Ottawa, Canada K1N 6N5

**Keywords:** *Parus major*, producer–scrounger games, social network analysis, social learning, social foraging, animal personality

## Abstract

There has been extensive game-theoretic modelling of conditions leading to equilibria of producer–scrounger dichotomies in groups. However there is a surprising paucity of experimental evidence in wild populations. Here, we examine producer–scrounger games in five subpopulations of birds feeding at a socially learnt foraging task. Over four weeks, a bimodal distribution of producers and scroungers emerged in all areas, with pronounced and consistent individual tactic specialization persisting over 3 years. Tactics were unrelated to exploratory personality, but correlated with latency to contact and learn the foraging task, with the late arrivers and slower learners more likely to adopt the scrounging role. Additionally, the social environment was also important: at the broad scale, larger subpopulations with a higher social density contained proportionally more scroungers, while within subpopulations scroungers tended to be central in the social network and be observed in larger foraging flocks. This study thus provides a rare example of a stable, dimorphic distribution of producer–scrounger tactics in a wild population. It further gives support across multiple scales for a major prediction of social foraging theory; that the frequency of scroungers increases with group size.

## 1. Introduction

Social animals can often choose among various strategies to obtain resources. One such strategy is scrounging, in which individuals exploit the time and energy investment of others rather than investing in finding or processing resources themselves. Exploitative strategies have been evidenced in several biological contexts, from mating (sneak copulations [1]), parental care (e.g. brood parasitism [2]), to foraging [3]. Of these, food scrounging is considered to be almost ubiquitous among social animals. It can take many forms, from direct theft (kleptoparasitism: [4]), to joining food patches discovered by others [5]. Such scrounging can strongly impact mean intake rate of group foragers and can thus potentially regulate group density and composition at multiple scales [6].

Fundamentally, producer–scrounger (P-S) models predict that social foraging tactics should be frequency-dependent, occurring with an equilibrium frequency in a given population [3]. Such theoretical modelling has generally not predicted ‘how’ this frequency of scroungers should be attained; i.e. with all individual scrounging at the same evolutionary stable strategy level (monomorphic population), or as various mixtures of individuals with different specialized tactics in a polymorphic population [5]. However in either case, evidence from previous research has suggested that individuals will tend to differ consistently in the degree to which they play each tactic (reviewed in [5,7]).

Such individual consistency is often related to state-based variables that change the relative pay-off of tactics for each individual; for example dominant individuals tend to adopt the scrounging role if scrounging involves kleptoparasitism or aggressive displacement [8–10]. There is also evidence for a link between personality traits and scrounging. For example, in captive barnacle geese (*Branta leucopsis*), ‘shy’ individuals scrounged more [11], a result the authors suggested might be due to a tendency for ‘bold’ individuals to be more active and less social, leading to a tendency to produce [11]. Yet other studies have found contrasting patterns between scrounging and personality [12–15], suggesting that the directionality of this relationship may be context-dependent. Finally, individual differences in P-S tactics can be driven by variation in learning ability if playing either tactic involves a skill component [16,17]. However, in any case, persistent individual differences in scrounging have largely been established using relatively short-term experiments in stable groups [18]. The extent to which wild individuals in naturally mixing groups consistently differ in their P-S tactics thus remains to be determined.

The equilibrium frequency of P-S tactics in a given population should also be dependent on various ecological scenarios [19,20]. In particular, the link between group size and P-S frequencies has received considerable theoretical attention, with larger groups predicted to support a higher frequency of scrounging [20–22]. Experimental manipulations have demonstrated that this effect can result from an adjustment in scrounging propensity by flexible group members (phenotypic flexibility) [23,24]. However an alternative, yet unexamined, way in which there could be covariation between group size and scrounging is by ‘reshuffling’ of individuals; for example, scroungers might choose to join larger groups or move between groups more often. This could additionally lead to a broader link between sociality and scrounging, if it results in scroungers inhabiting more central social network positions. Scroungers have been shown to maintain central spatial position in their groups to maximize access to food discoveries [25,26]; yet the extension to population-level patterns through social network analysis has, to the best of our knowledge, never been investigated.

Here, we examine P-S games in five subpopulations of great tits (*Parus major*) foraging at a socially learnt puzzle-box in the wild [27], where scrounging can occur either by displacement of the solving bird or a visit to the solved task immediately (1 s) after the solving bird. We first assess the overall distribution of P-S tactics in each subpopulation, and examine individual consistency in tactic choice over both short (one month)- and long-term (3 years) periods. We then test a set of candidate individual predictors of scrounging behaviour, including sex, age, learning speed and the personality trait ‘exploration behaviour’. Finally, we explore the relationship between sociability and scrounging at multiple scales. At the broadest scale, we ask whether there are proportionally more scrounging-specialist individuals in larger subpopulations. Within subpopulations, we compare an individual's propensity to scrounge with independently obtained measures of its sociability, including average group size and social network centrality. Our study thus provides a comprehensive examination of the individual, social and group-level factors driving the distribution of scrounging behaviour in a wild population of socially foraging birds.

## 2. Methods

### (a) Study system

The study was conducted in a population of great tits (*Parus major*) at Wytham Woods in the UK (51°46′ N, 01°20′ W), over three winters from November 2012 to March 2015. Wytham Woods is a 385 ha area of mixed broadleaf woodland, where from autumn to winter, birds form loose flocks of unrelated individuals [28], with groups aggregating to exploit patchy food sources. The population here has been the subject of a long-term study; all resident great tits are caught as chicks or breeding adults and fitted with a British Trust for Ornithology metal leg ring and a plastic leg ring encasing a uniquely identifiable passive integrated transponder (PIT) tag (IB Technology, Glenfield, UK). Mist-netting additionally targets individuals immigrating into the population, with these birds sexed and aged upon capture.

### (b) Personality assays

Individuals were assayed for the personality trait of *exploration behaviour in a novel environment*. This trait forms part of a reactive-proactive axis, contrasting shy, slow-exploring individuals with bolder, fast-exploring individuals [29]. Behavioural assays have been ongoing in this population since 2005 [30], and we followed existing methods (see [30,31] for more detail). Individuals were caught with mist-nets outside the main experimental periods at various times between October-March and taken into captivity. The following morning birds were assayed individually in a novel environment containing five artificial trees. Twelve types of behavioural observations were recorded over an 8 min period, including flight number, flight durations, hop number, substrate used and area explored. All birds were then released at site of capture. In principal component (PC) analysis, PC1 explained 45% of the variation, and the square-root of PC1 was used in a general linear model (GLM) with observation number, time of year and individual identity as fixed effects. The predicted estimates of individual intercepts from this model were used to create a single exploration score for each individual (*repeatability estimate* = 0.35; see [30] for more information).

### (c) Social foraging experiment

A social learning and foraging experiment was conducted in five relatively isolated subpopulations across the woodland, in four-week periods between January and March 2013 for two subpopulations (T2 and T3), and in four-week periods between December 2013 and February 2014 for three subpopulations (T1, T4, T5) [27]. Prior to the first week of data collection, two males were caught from each subpopulation and trained in captivity to solve a novel puzzle-box, then released to act as the initial demonstrators for this behaviour. Three such puzzle-boxes were then installed 250 m apart in each subpopulation, continuously operating from dawn on Monday to dusk on Friday for a total of 20 days. These puzzle-boxes consisted of an opaque plastic box with a perch in front of a door that could slide in either direction to gain access to a concealed feeder. This box contained approximately 500 mealworms, and was refilled up to twice daily as necessary. Live mealworms are a highly preferred food type, and as the prey was alive, birds typically extracted one worm and then carried it away to kill and eat it [27]. Once installed, puzzle-boxes were surrounded by a 1 × 1 m cage with a 5 × 5 cm mesh that prevented access by larger non-target species (see [27] for more detail).

All puzzle-boxes also contained a printed circuit board (Stickman Technology, Southampton, UK), with the perch functioning as an RFID antenna registering the identity, visit duration and action of each visiting individual. A ‘*solve*’ was recorded when a visiting individual opened the puzzle-box door to access the feeder, here classed as a ‘producer’. One second after the bird departed, the door closed. If a different individual visited the puzzle-box within this second, then a ‘scrounge’ was recorded, as this individual was assumed to have taken food (video observations more than 30 h confirmed that birds took food items from 92% of visits to open doors; similarly for producers or scroungers). The door closed 1 s after this scrounging individual departed; however if an additional tagged bird visited within this time, a second ‘scrounge’ was allowed in the same manner. The door began to shut upon detection of a third scrounging individual, thus preventing more than three possible *scrounges* per *solve*. In all areas the solving behaviour spread rapidly, with 68–83% (*n* = 37–96 per subpopulation) of resident individuals solving at least once, and with 7945–12411 rewarded visits to puzzle-boxes per subpopulation (an average of 170 rewarded visits per puzzle-box per day); for more detail see [27].

Given mortality rates in this population, we expected approximately 40% of individuals to overlap between consecutive years [32]. This was the case, and birds recorded in more than 1 year also clearly retained a memory of how to solve the puzzle-box [27]. After the initial experimental periods, puzzle-boxes were therefore reinstalled at two subpopulations (T2, T3) for an additional 5 days in December 2013 (see [27]), and in all five subpopulations for an additional 20 days over December 2014 – February 2015. Data on individuals' visits to puzzle-boxes were thus repeatedly sampled over 2–3 years depending on subpopulation: throughout the text each of these experimental periods in a given year/subpopulation is referred to as a ‘replicate’.

### (d) Social networks

During the initial experimental periods, eight sunflower-seed bird-feeding stations were deployed around each subpopulation in an approximate 250 × 250 m grid. Each feeding station had two openly accessible feeding holes fitted with RFID antennae and data-logging software that registered all visits by PIT-tagged individuals, scanning for PIT tags every 1/16 s. These stations opened from dawn to dusk on Saturday and Sunday for weekends within and surrounding the experiment for a total of 10 days. They thus provided an independent observation of the spatio-temporal flocking patterns of birds. Therefore, to assess individuals' sociability, we identified social groups in feeder visits by using a Gaussian mixture model that detected naturally occurring ‘gathering events’ in the data without imposing arbitrary assumptions about group size or composition [33,34]. Associations were calculated using a gambit of the group approach, where individuals were given a link if observed in the same gathering event [35]. These associations were then scaled from 0 (never observed in the same group) to 1 (always observed in the same group) [36]. All social analyses were conducted in R, using the packages *asnipe* [37], *sna* [38] and *igraph* [39]. The five resulting social networks were demonstrated in a previous study to contain preferred and avoided relationships, and were an important predictor of the transmission of information about how to solve the puzzle-box [27].

### (e) Statistical analysis

Scrounging and solving events were collated for each individual. A longitudinal clustering algorithm [39] then fitted the data for the relative proportion of events that were scrounges for each individual over cumulative 2 h time periods. This partitions trajectories into behavioural clusters, and was used to test for a bimodal distribution of social foraging strategies across all individuals. This method provides a more comprehensive test for multi-model distributions in longitudinal data, and was implemented in R [40] using the package *kml3d* [41].

To examine consistency in individuals' social foraging strategies, we calculated intraclass correlation coefficients (ICC) for each four-week replicate and between years; this gives a measure of the total variation that is reproducible among repeated measure of the same individual [42]. Linear mixed models were used in the R package *MCMCglmm* [43] to calculate estimates and their associated significance, with Markov chain Monte Carlo sampling using restricted maximum likelihoods and default priors. All rewarded events (whether *solve* or *scrounge*) for all individuals were included in the model, with date and replicate identity (four-week experimental period, a combination of time and location), included as fixed effects. Repeatability was calculated across years using the same method, but including year and subpopulation as fixed effects. Repeatabilities were then estimated by dividing the variance of the individual random effect by the sum of the variances of individual-level and random error [42].

Five individual-level variables were considered as potential predictors of P-S tactics: age, sex, exploratory personality, time of first contact and latency to learn after first contact. Age (first year or adult) was known for 586 birds (100%), sex for 563 birds (96%) and personality for 121 birds (21%). Time of first contact was defined as the latency (s) from the beginning of the experiment until an individual was first detected on the puzzle-box, excluding 18.00–6.00 and weekends when puzzle-boxes were not available. This detection was neither a scrounge or solve, but could be potentially followed by either. Latency to learn was then calculated as the cumulative number of seconds after this first detection that an individual spent at the closed door of puzzle-box before it solved (often across multiple occasions). This measure is similar to that used in a study investigating problem-solving efficiency in great tits [44], but here the task is socially learnt, so there is also likely to be a social component to this score. All individual-level measures were unrelated to each other except for first-contact time and age, which were moderately correlated. Therefore, the total number of scrounging and solving events for each individual in a given replicate was compared in a general linear mixed model (GLMM; R package *lme4*) against six fixed effects: age, sex, personality, first contact time, latency to learn; including total number of rewarded visits as a fixed effect for control, and including replicate ID (four-week experimental period in a subpopulation) as a random intercept. Data across the 3 years of data collection were included, but analysis was restricted for each individual to its first observed sampling period to control for any effect of accumulated experience across years. Finally, all models were restricted to individuals observed ≥50 times in order to get a good estimate of their overall behaviour. However, all results were robust to variation in this threshold (results repeated at ≥10, ≥20).

The link between sociability and scrounging was examined at two levels; at the replicate level (between different time periods in different subpopulations) and within replicates (between individuals within four-week experimental periods). For the former, the proportion of individuals that were identified as scroungers in the longitudinal clustering model (see above) was regressed against subpopulation size, where subpopulation size was a count of all individuals that were detected on puzzle-boxes during a given replicate. Subpopulation size varied from *n* = 40 (T3/Yr2; 27 with ≥50 visits) to *n* = 102 (T3/Yr1; 77 with ≥50 visits). For the latter, we compared individual differences in their average group size and in three social network metrics. First, we identified all gathering events at the openly accessible data-logging sunflower feeders in which a focal individual occurred. These gathering events are considered to be equivalent to foraging flocks and are highly fission-fusion [28]. Overall, average group size was 7.9, however individuals varied in their average group sizes from 1.3–15.5. Second, we calculated three commonly used measures of social network centrality for each individual: association strength (weighted degree), unweighted degree and betweenness [45]. Association strength is the total interaction rate for a given node with all other nodes, and is a good measure of overall sociability. Unweighted degree is a count of all others that a given individual was observed feeding with, and is a measure of gregariousness. Finally, betweenness is the number of shortest paths between all nodes that pass through the focal individual, and reflects the propensity of individuals to move between groups [45]. Previous work in this population has demonstrated that individuals are repeatable in these metrics within and between years [46].

Scrounging propensity was compared to social predictors in a GLMM; this was as for the other, non-social individual-level predictors, but excluding exploratory personality. The model then included an additional fixed effect of either average group size, association strength, degree or betweenness. These measures are correlated to varying degrees, and so separate models were run for each metric. Therefore, each model compared the total number of scrounging and solving events for each individual against six fixed effects: age, sex, first contact time, latency to learn, total number of rewarded visits and either average group size, strength, degree or betweenness, with replicate ID as a random intercept. Individual scores are not independent of each other in a social network, and so significance was calculated using randomisations [46]. Using the R package *asnipe*, we conducted 10 000 permutations of the data stream derived from social network data-loggers, controlling for date and location of the observations. A GLMM was run after each permutation for each social measure, giving a distribution of parameter estimates. This was compared to the parameters derived from the actual data, with significance assigned if the values fell outside the 95% range of estimates. Conducting randomisations at this local level allows individual differences to be disentangled from any effect of population size or density [37].

## 3. Results

### (a) Identifying producer–scrounger behaviour

**Figure 1.**
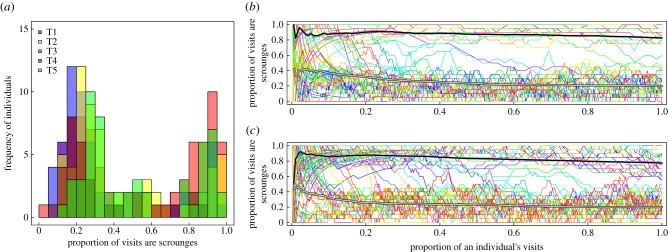
(*a*) Distribution of scrounging behaviour in individuals across subpopulations T1–T5 in an initial four-week observation period. Bars are semi-transparent and overlaid to show all replicates, and only individuals with ≥50 visits are shown. (*b*) Example of individual longitudinal trajectories from T1, showing a different coloured line for each individual of the running mean of the proportion of their last 20 rewarded visits that were scrounges, plotted proportional to their total rewarded visit count; again only individuals with ≥50 visits are shown. A longitudinal clustering algorithm identifies two distinct clusters, shown in black (‘scroungers’) and grey (‘producers’). (*c*) Example of individual longitudinal trajectories from T3. Note that in both (*b*) and (*c*) switches are infrequent.

A total of 375 (T1 = 45, T2 = 64, T3 = 102, T4 = 36, T5 = 95) great tits made rewarded visits to puzzle-boxes in the five subpopulations of the initial experiment (259 with ≥ 50 visits), with 586 great tits recorded across 3 years of data collection (430 with ≥ 50 visits). Of these, only nine birds never scrounged (1 with ≥ 50 visits) and 40 birds specialized entirely on scrounging (2 with ≥ 50 visits), suggesting that: (i) scrounging did not prevent social learning, and (ii) almost all individuals that made numerous visits at least partly engaged in both tactics. Puzzle-boxes were used frequently, with 176 107 rewarded visits across all three winters. A mean of 33% (26–40% range of replicate means) of these rewarded visits were scrounging events in the initial experiment (figure 1*a*). This declined slightly in the second (24%, 16–32%) and third winters (23%, 21–24%). A clustering algorithm partitioned data into two clear groups with different mean scrounging rates in all areas (average cluster means: 0.27, 0.86). This bimodality persisted across all winters in all areas that were observed (cluster means; Yr2: 0.25, 0.87; Yr3: 0.16, 0.9). A longitudinal clustering algorithm was therefore used to categorize individual trajectories into one of these two groups (e.g. figure 1*b*,*c*).

### (b) Individual consistency in producer–scrounger tactics

In the first experimental period in which they were observed, individuals made on average 173 rewarded visits to puzzle-boxes, with birds favouring the producer tactic tending to make more rewarded visits (GLMM: *z*_372_ = −81.98, *p* < 0.001). Across this period, birds were consistent in the degree to which they used both strategies: *R* = 0.50, 95% CI 0.46–0.53 (figure 1*b*,*c*). A total of 154 individuals were also observed in more than 1 year, with 30 individuals observed in all 3 years of sampling. Birds were similarly repeatable in their tactic use across years (*R* = 0.51, 95% CI 0.48–0.55), although individuals tended to move towards producing over time (100% of ‘producers’ (*n* = 88) and 54.2% of ‘scroungers’ (*n* = 72) used producing tactics in later years).

### (c) Individual-level determinants of tactic choice

**Figure 2.**
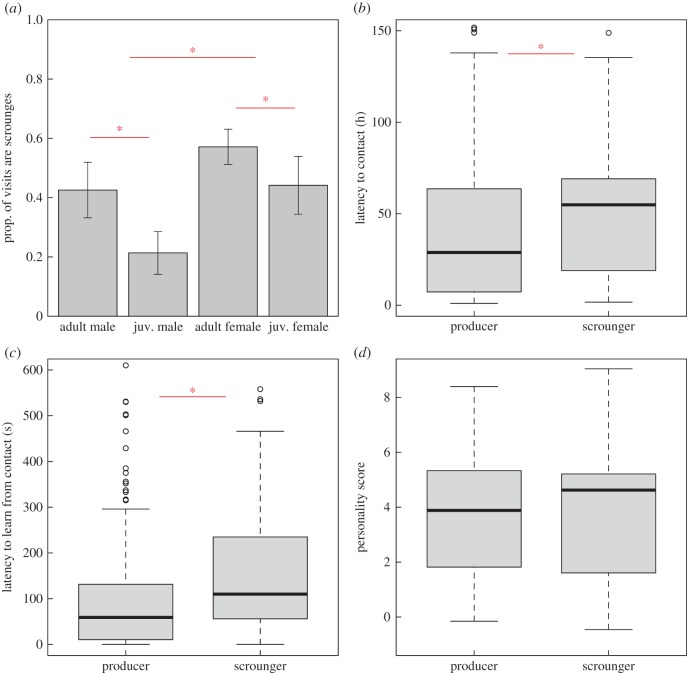
Individual (non-social) predictors of tactic choice, showing all birds with ≥50 visits. Asterisks highlight significant differences. (*a*) Adults and females were more likely than males and juveniles to be classed as scroungers in a longitudinal clustering model. Vertical bars show standard error. (*b*) Birds that arrived later in the experimental period were more likely to adopt the scrounging role. (*c*) Scroungers also tended to have a longer latency to learn than producers. (*d*) There was no relationship between scrounging and personality score. Horizontal lines show median in (*b*), (*c*) and (*d*). (Online version in colour.)

Adults tended to scrounge more than first-year birds, although the effect was stronger in males (table 1*a*; males—GLMM: *z*_221_ = −7.59, *p* < 0.001), figure 2*a*. Across the sexes, females scrounged more than males (table 1*a*; figure 2*a*). While we did not measure dominance directly, in great tits older males are dominant over young males and all males are dominant over females [47]. It therefore seems unlikely that dominance primarily determined scrounging tendencies, although it is possible that dominance interactions occurred primarily within each sex. Time of first contact was also related to tactic choice, with individuals that arrived later more likely to adopt the scrounging role (table 1; figure 2*b*). This carried through to ‘learning speed’, with individuals that had a longer latency between contacting and solving the puzzle-box also tending to adopt the scrounging role (table 1*a*; figure 2*c*). In total, 121 individuals were assayed for exploratory personality (T1 = 29, T2 = 31, T3 = 35, T4 = 12, T5 = 14). When we included it in the model, we found no relationship between personality and foraging strategy (table 1*a*; figure 2*d*).

**Table 1.** GLMM model showing individual and social predictors of scrounging propensity. (Significant *p-*values are indicated in italics, *n* = 372 for all individual predicators other than exploration personality, where *n* = 84. *n* = 224 for all social network metrics, *n* = 274 for average group size. For more detail see the electronic supplementary material, table S1.)

	coefficient	s.e.	*p*
(*a*) individual predictors			
age (adult/first year)	−0.051	0.017	*0.003*
sex (F/M)	−0.400	0.016	*<0.001*
time of first contact	−0.097	0.008	*<0.001*
latency to learn	0.169	0.005	*<0.001*
exploration behaviour	−0.003	0.009	0.67
(*b*) social predictors			
average group size	0.159	0.005	*<0.001*
betweenness centrality	0.001	0.002	0.26
unweighted degree centrality	1.066	0.039	*0.003*
association strength	0.253	0.008	*<0.001*

### (d) Social determinants of tactic choice

**Figure 3.**
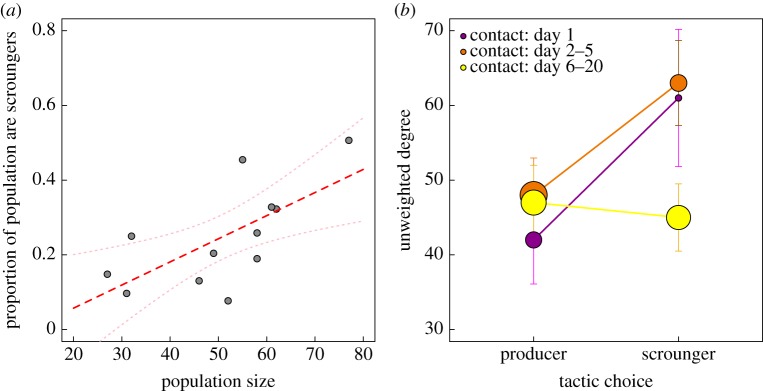
Social predictors of tactic choice, showing all birds with ≥50 visits. (*a*) The proportion of birds that were classed as scroungers in a longitudinal clustering model was higher in replicates with higher population sizes. Lines show linear model fit and 95% CI (*p* = 0.01, *R*^2^ = 0.39). (*b*) Unweighted degree was higher for birds classed as scroungers in a longitudinal clustering model. However sociality interacted with the time of contact in the experimental period; birds that visited on day 1 (*magenta*) and in the first week (day 2–5, *orange*) showed a positive relationship between degree centrality and likelihood of scrounging, but birds that arrived later (day 6–20, *yellow*) showed no relationship. Points represent median and size is relative to the number of individuals in each category (day 1, *n* = 46; day 2–5, *n* = 89, day 6–20, *n* = 100), vertical bars show 95% CI. (Online version in colour.)

Across all experimental periods in all subpopulations, the overall proportion of scrounging-specialist individuals was correlated with the total number of individuals visiting puzzle-boxes, suggesting a positive relationship between subpopulation size and frequency of scrounging (controlling for year; linear model: *t*_12_ = 3.09, *p* = 0.01, *R*^2^ = 0.60, figure 3*a*). Within subpopulations, average group size and three social network metrics were compared against strategy choice. There was no clear relationship between an individual's betweenness and scrounging (table 1*b*). However there were significant relationships between scrounging and unweighted degree, association strength and average group size (table 1*b*; figure 2*d*; electronic supplementary material, figure S1). That all three measures were significant was not surprising, as in a ‘gambit of the group’ social network, individuals' group sizes limits the range of their association strength [45]; indeed average group size and association strength were highly correlated (Pearson's correlation: 0.74).

As data randomizations only permuted individuals within single day/location, we can further conclude that the relationship between scrounging and sociality was observed at the most local level, and was therefore additional to the effect of variation in subpopulation sizes. However an examination of the data did reveal an interaction between sociality and contact order (unweighted degree, GLM: *z*_224_ = 2.52, *p* = 0.01; association strength, GLM: *z*_224_ = 12.24, *p* < 0.001), with the link between tactic choice and network centrality not present for individuals that arrived in that last part of the cultural diffusion process (for example, figure 3*b*). In this latter period the frequency of solving events at puzzle-boxes is high, and so scrounging opportunities may be effectively unrestricted [27].

## 4. Discussion

By monitoring flocks of great tits feeding at foraging tasks, we show that individuals specialize on producing or scrounging, leading to a bimodal distribution of P-S behaviour in each of five subpopulations over 2–3 years measured. Individual specialization to a single tactic was stable even between years; a striking result when considering that birds had no exposure to the task in the intervening 9–12 months between sampling periods. Tactic specialization was influenced by several independent factors. First, females and adults were more likely to scrounge than males and juveniles. In a previous study in this population, females closely followed their mates to feeders [48] – it is possible that the sex differences observed here reflect a similar tolerance between mated pairs, with dominance determining the males' priority of access and hence tendency to produce [12]. Second, tactic choice was predicted by social factors. Between areas, the proportional frequency of scroungers was positively correlated with population density. Within areas, individuals that arrived earlier and learnt the task faster were less likely to scrounge, and scroungers tended to have higher network centrality and larger average group sizes.

The consistency in tactic choice observed in our experiment is in agreement with previous studies, where individuals have tended to be repeatable in their proportional use of scrounging, albeit over much shorter time periods [11,12,17,18,49]. Individual repeatability might reflect stability in the resources and social environment, with corresponding stability in the relative tactic pay-offs for each individual [18,50]. In our study, the resource availability was controlled by the automated foraging task, and remained unchanged. Additionally, despite their fission-fusion social dynamics, individuals show consistent preferences in their overall sociality and gregariousness [46]. It is therefore possible that individual consistency in tactic choice may have changed if the foraging task or social network were experimentally perturbed – this remains to be tested.

Interestingly, when individuals did change their behaviour between years this was always a switch from scrounging to producing. This within-individual trajectory is opposite to that across individuals, where older birds are more likely to scrounge. P-S theory gives no indication as to what direction individuals should shift. However birds were foraging on a resource that represented a learnt skill [27], at which they became more efficient over time (L. M. Aplin 2013, unpublished observation). It seems possible that as some individuals became more efficient at producing, the relative costs of the tactics re-balanced, leading to an eventual behavioural shift [15,51]. This may be further exaggerated between years, when there is an initial period (before juveniles acquire the skill) in which there are relatively few surviving producers to scrounge from [27]. In this way, while scrounging does not directly promote social learning (unlike in [52]), it may provide a back-route into skill acquisition, keeping slower learners engaged with the foraging task and thus providing a ‘grace’ period for eventual mastery of the behaviour.

In investigating the social factors underlying P-S behaviour, our study provides evidence for a correlation between sociality and scrounging at multiple scales. At the broadest scale, the relative frequency of scroungers was higher in replicates with higher population density. Within subpopulations, social network analysis revealed a positive relationship between sociability and scrounging, whereby scroungers were more likely to occur in larger group sizes, have a greater total number of foraging associates, and spend more of their foraging time with other individuals. This is consistent with theoretical predictions that larger group sizes should contain a higher frequency of scroungers [20], and uniquely demonstrates the generality of this effect, from individual decision-making to population-level patterns. Finally, our results represent a novel mechanism by which this relationship occurs: more sociable individuals enjoy greater exposure to scrounging opportunities, and/or scroungers choose to join larger flocks. Given that the relationship disappeared late in the cultural diffusion process when scrounging opportunities were almost universal [27], it seems most likely that the causality goes in the first suggested direction.

Two captive studies also support a relationship between group size and scrounging: in social spiders (*Australomisidia ergandros*), larger groups had a higher scrounger to producer ratio, mediated by a shift in the relative frequency of tactic-specialized individuals [23]; while in nutmeg mannikins (*Lonchura punctulata*) birds increased their use of scrounging with increasing group size [24]. We extend this evidence to a wild population, and demonstrate that the link between sociality and scrounging is robust to fission-fusion dynamics [28], suggesting that stable groups are not necessary for this pattern to occur. Finally, our results suggest that a correlation between sociality and scrounging in fission-fusion populations can still be achieved with tactic-specialized individuals as long as individuals differ consistently in their level of sociability (previously demonstrated in this population [46]), highlighting how interacting individuals can experience different social environments.

Overall, our study reveals that wild great tits maintain a strong and consistent tactic-specialization when foraging at a socially learnt resource, resulting in a bimodal distribution of P-S strategies across the population. This is, to our knowledge, the first such reported in a wild population. Indeed, social foraging theory predicts that behavioural polymorphisms, with individuals specializing on ‘pure’ strategies (either producing or scrounging) will only arise under limited conditions [20,53]. This includes when there are fixed genetic differences between individuals, or when phenotypic differences between individuals lead to differences in the pay-offs for each strategy [20]. One hypothesized route by which this may occur is via heritable variation in personality, for example in geese less exploratory individuals are more likely to adopt scrounging roles [11]. However this relationship is not universally supported [12,14], and could potentially be mediated by correlates of personality types (e.g. differences in arrival order) [54]. Our study did not find any evidence for a relationship between the personality trait ‘exploration behaviour’ and scrounging. This particular personality trait is very well studied in great tits [30,55]. Given our comparatively large sample size, it seems likely that this is a true negative result and inherent personality differences do not underlie tactic choice in this context.

Rather we would suggest that behavioural polymorphisms could also arise when there is a significant skill-learning component to one or both tactics. This could lead to a positive feedback loop where experience on one tactic makes it more profitable/less costly to perform in future [16,17,56]. This is consistent with our finding that arrival time, learning speed and social connectivity all predict tactic specialization. Birds in areas of higher local density, more social individuals, and late arrivals to the foraging task all experienced more exposure to producers, giving them more opportunity to initially engage in scrounging. It seems likely that their tendency to continue scrounging was further influenced by individual differences in learning speed. This variation in learning latency would change the relative costs and benefits of each strategy for each individual, exaggerating positive feedback loops further; thus leading to tactic specialization and an eventual bimodal distribution of P-S behaviour at the population level.

## Supplementary Material

Extra information on model parameters

## Data accessibility

Data are available from the Dryad Digital Repository: http://dx.doi.org/10.5061/dryad.n7c42 [57].
