# Supplementary material for: Stable producer–scrounger dynamics in wild birds: sociability and learning speed covary with scrounging behaviour
Source: Proc Biol Sci. 2017 Apr 12;284(1852):20162872. doi: 10.1098/rspb.2016.2872 (PMC5394662; doi:10.1098/rspb.2016.2872)
Supplement: Extra information on model parameters [file rspb20162872supp1.docx]

**SUPPLEMENTARY MATERIAL**

**Table S1.** Table showing model structure of GLMM model comparing individual (non-social) predictors with scrounging propensity. *N=372* in all cases but for personality, where *N=84.* Some variables were log transformed in the model (time of first contact, latency to learn, total no. of visits)

|  | *Coefficient* | *s.e.* | *z-score* | *P* |
| --- | --- | --- | --- | --- |
| *(a) Fixed effects* |  |  |  |  |
| Age (Adult/First Yr.) | -0.051 | 0.017 | -2.97 | **0.003** |
| Sex (F/M) | -0.400 | 0.016 | 24.61 | **<0.001** |
| Time of First Contact | -0.097 | 0.008 | 12.56 | **<0.001** |
| Latency to Learn | 0.169 | 0.005 | 35.73 | **<0.001** |
| Exploration Behaviour | -0.003 | 0.009 | -0.42 | 0.67 |
| Total no. of Visits | -1.196 | 0.005 | 81.98 | **<0.001** |
| *(b) Random effects* |  |  | *Var.* | *Std. Dev.* |
| Replicate ID (12 levels) |  |  | 0.206 | 0.453 |

**Figure S1.**  Permutation tests showing comparisons between the observed estimates of four social measures from the generalized linear mixed model (red vertical lines) with distribution of estimates calculated from spatially constrained null models that randomise social data within sampling period and within location. Results show a significant difference for all measures other than betweenness.
